# Headphone Audio in Training Systems or Systems That Convey Important Sound Information

**DOI:** 10.3390/ijerph19052579

**Published:** 2022-02-23

**Authors:** Rafal Mlynski

**Affiliations:** Department of Vibroacoustic Hazards, Central Institute for Labour Protection—National Research Institute, Czerniakowska 16, 00-701 Warsaw, Poland; rmlynski@ciop.pl

**Keywords:** sound, noise, headphones, earphones, sound pressure level, occupational exposure, hearing, audio strip

## Abstract

In the work environment, miniature electroacoustic transducers are often used in communication, for the transmission of warning signals or during training. They can be used in headphones or mounted in personal protective equipment. It is often important to reproduce sounds accurately. The purpose of this work was to assess audio strips by comparing the frequency response of the signal in the electrical outputs of six common-purpose devices. Based on the risk of hearing damage, the level of noise exposure was assessed. The following headphones were investigated: low-budget closed-back, open-back for instant messengers, open-back for music, and in-ear. A head and torso simulator with a transfer function was used. The most uniform shape of the frequency response of the signal at the electrical outputs was found to be in smartphones. Sound cards integrated into laptop motherboards had highly unequal characteristics (up to 23 dB). In the case of one of the laptops, the upper range of the transmitted frequencies was limited to the 12,500 Hz band. An external sound card or wireless headphones can improve the situation. In the worst-case scenario, i.e., rock music, the listening time was limited to 2 h and 18 min.

## 1. Introduction

In many situations, relevant information—and in certain situations, warning signals—are provided to employees by means of sound played over an electroacoustic transducer located close to the ear. This can be in the form of headphones, e.g., for employees communicating with colleagues or customers. It can also be in the form of a miniature speaker installed in personal protective equipment (PPE), such as a hearing protector or helmet, as described in a previous paper [1]. When providing direct audio information, support and training tools for employees are increasingly delivered in the form of presentations, applications, and videos intended to be run on common-purpose devices. The training often contains information on how to properly use personal protective equipment. Training can also be addressed to other audiences, e.g., persons with vision disabilities, as presented in a previous paper [2].

Sound is an integral part of these solutions. When the information contained in the sound is relevant, such as in the training process, it is also important that the sound present in real-life surroundings is reflected as closely as possible. In order to reach individuals in a manner that best suits the real conditions, teams preparing materials for training should be aware of the capabilities and limitations of the equipment potentially available to those who will use such tools. Another important consideration when using sound communication tools is to maintain safe conditions during their use. It is essential that exposure to sound is safe. Thus, knowledge of the level of sound generated during playback is important.

The parameters of sound played through headphones were subjected to a series of tests [3] preceding the 2009 publication of Commission Decision 2009/490/EC [4], which recommended that European standards should include the requirement to limit the sound level produced by personal music players. This limitation is intended to prevent hearing damage. The Decision states that “at 80 dB(A) exposure time shall be limited to 40 h/week, whereas at 89 dB(A) exposure time shall be limited to 5 h/week”. The limit of 40 h/week, according to the principles applied in assessing noise exposure in the work environment, corresponds to 8 h per workday. The value of 89 dB results from the principle of exposure time being halved, with a 3 dB increase in the parameter characterizing the energy properties of the noise. Details on the issues covered by the Decision, as well as the basis for its provisions, are included in the SCENIHR report [3].

The limitation of noise level with regard to equipment used during work should also be considered in conjunction with regulations related to the work environment. In Poland, the criterion value of the A-weighted noise exposure level, normalized to an 8 h working day (daily noise exposure level), is 85 dB [5]. Hearing protection can also be based on data on the standard for estimating hearing damage caused by noise [6]; from this, it can be concluded that the real criterion value, due to the absence of an impact on hearing, should be 80 dB. It is worth mentioning that this is also the lower exposure action value established in the relevant Directive [7], and exceeding it mandates certain actions to reduce the risk of noise exposure. Unfortunately, it has been reported that in a number of situations, exposure to sound generated by headphones exceeds those limits. 

In the same year that the Decision was published, one paper reported that as many as 30% of a group of 70 young adults included in a study listened to music beyond the safe limit of 80 dB of the A-weighted equivalent sound pressure level over 8 h, as specified by the Ministry of Environment and Forests in India [8]. Another paper reported that 58.2% of college students, aged 18–53 years, who used personal music players exceeded the 8 dB A-weighted noise exposure level, normalized to an 8 h working day [9]. Another paper measured the A-weighted equivalent sound pressure level for 61 people listening to music at 73 to 102 dB, and stated that listeners were divided into those who listened to music less frequently at a lower, safer volume, and those who may suffer hearing damage over time [10]. In another study, 7 out of 12 participants listened to music using a personal music player at a volume nearly exceeding an A-weighted sound pressure level of 80 dB [11]. 

This problem is also illustrated by newer data. One paper in 2017 [12] reported that while 80% of young people listened to their favorite music at an A-weighted equivalent sound pressure level below 85 dB, 10% preferred listening at 85 to 90 dB, and another 10% at 90 to 100 dB. Some papers pay attention to the noise parameters associated with the use of headphones, and others to the possible harmful effects on hearing of the sound generated by headphones. In a study assessing the impact of different types of headphones (closed-back, semi-open, open-back, and in-ear) on hearing loss, and analyzing the results of measuring hearing loss among 81 young people aged 16 to 25 years, it was found that almost 15% of those tested experienced a hearing threshold shift, especially at 4 kHz. This means that they had the first symptoms of permanent hearing damage [13].

Sound parameters are also particularly important for designing audio safety signals to be used in the presence of noise. When designing such signalling, it is necessary to have knowledge of the sound pressure level in individual frequency bands [14]. The selected parameters of headphones are included in the specifications provided by the manufacturer. With regard to using headphones in training systems or systems that convey important audio information, the properties of the strips playing back the sound, i.e., the device that converts sound from digital to analogue form and controls the headphones, are important. The aim of this work was to assess the strips playing back sound in headphones connected to the output of a sound card integrated into a laptop, as a common-purpose device, by studying their frequency response. The level of noise exposure was assessed under the assumption of theoretical extremely unfavorable conditions for the use of such audio strips in real-life circumstances, with the sound played back over headphones. The purpose of the study was to compare the frequency response of the signal of the electrical output of six devices converting audio from digital to analogue and controlling the headphones. The devices were: sound cards built into two laptops, two personal devices, a USB digital interface, and a digital–analogue wireless headphone transducer.

## 2. Materials and Methods

### 2.1. Audio Playback Strips and Headphones Used in the Tests

The audio material intended for use in training is most often transmitted via equipment that enables sound to be played over headphones connected, for instance, to a PC or smartphone. Headphones connected to consumer devices are also used by employees who communicate with each other or with clients. Miniature electroacoustic transducers, similar to those installed in consumer headphones, are also mounted in the cups of earmuffs equipped with electronic circuits. Frequency response studies take into account the audio strip, which consists of a sound card built into a laptop and connected to headphones. In the discussed solutions, headphones very often receive signals from a common-purpose device such as a laptop.

Headphones of different designs and prices were selected for the study, assuming that they represented exemplary, commonly used equipment. The aim of this work was not to characterize the devices available on the market; as long as the correctness of the equipment was verified, the model names of devices and headphones included in the research were not mentioned. The headphones selected for testing can be characterized as follows: (1) circumaural with a closed-back structure and a microphone on a cable, priced at about EUR 15 (low-budget headphones); (2) supra-aural with an open-back structure, dedicated to instant messengers, with a microphone on a wire, priced at about EUR 65 (headphones for instant messengers); (3) circumaural with an open-back structure, dedicated to listening to music, no microphone, priced at about EUR 150 (headphones for music); and (4) in-ear with a microphone on a cable, priced at about 9 EUR (in-ear headphones).

As mentioned in the introduction, the characteristics of sound coming from the audio equipment to the listener are influenced by each component of the strip transmitting the sound. It is, first, a device that converts sound from digital to analogue form (i.e., a sound player), and second, a headphone containing an electroacoustic transducer. The device shapes the signal before it is delivered to the headphones, and has a significant impact on what reaches the listener. Even the best-quality headphones will not be able to satisfactorily reflect the sound being played if the electrical signal fed to the headphones is distorted. Thus, the measured frequency response of the entire audio strip (the sound pressure level measured at the output of the signal playback over the headphones) is supplemented by the characteristics of the sound-shaping device (the electrical signal measured at the electrical outputs). 

**Figure 1:**
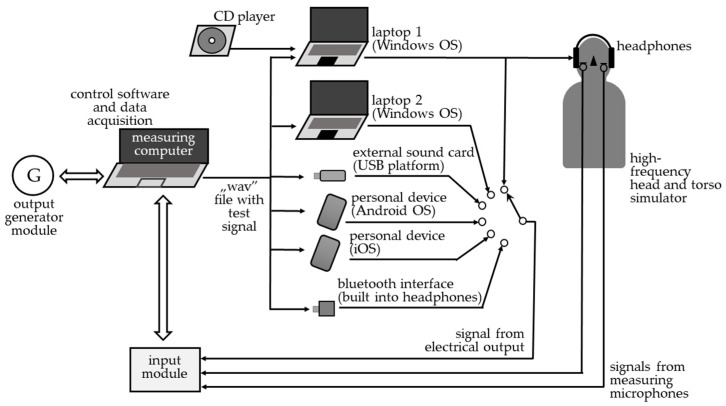
Method of conducting tests, including elements mentioned in Section 2.1, Section 2.2 and Section 2.3.

At the same time, in addition to the sound card built into a laptop operating with Windows OS (laptop 1), there was also an external digital interface, i.e., an external (portable) sound card communicating with the computer via a USB port (external sound card (USB platform)). For comparative purposes, the scope of measurements was extended, taking into account another model of sound card built into a laptop (from another manufacturer, operating with Windows OS (laptop 2)) and two personal devices, a smartphone/tablet, and wireless headphones, with data transmitted using the Bluetooth standard (or Bluetooth interface). One personal device was operating with Android OS, and the other with iOS. A diagram illustrating the test method is presented in Figure 1.

### 2.2. Measuring Equipment

**Figure 2:**
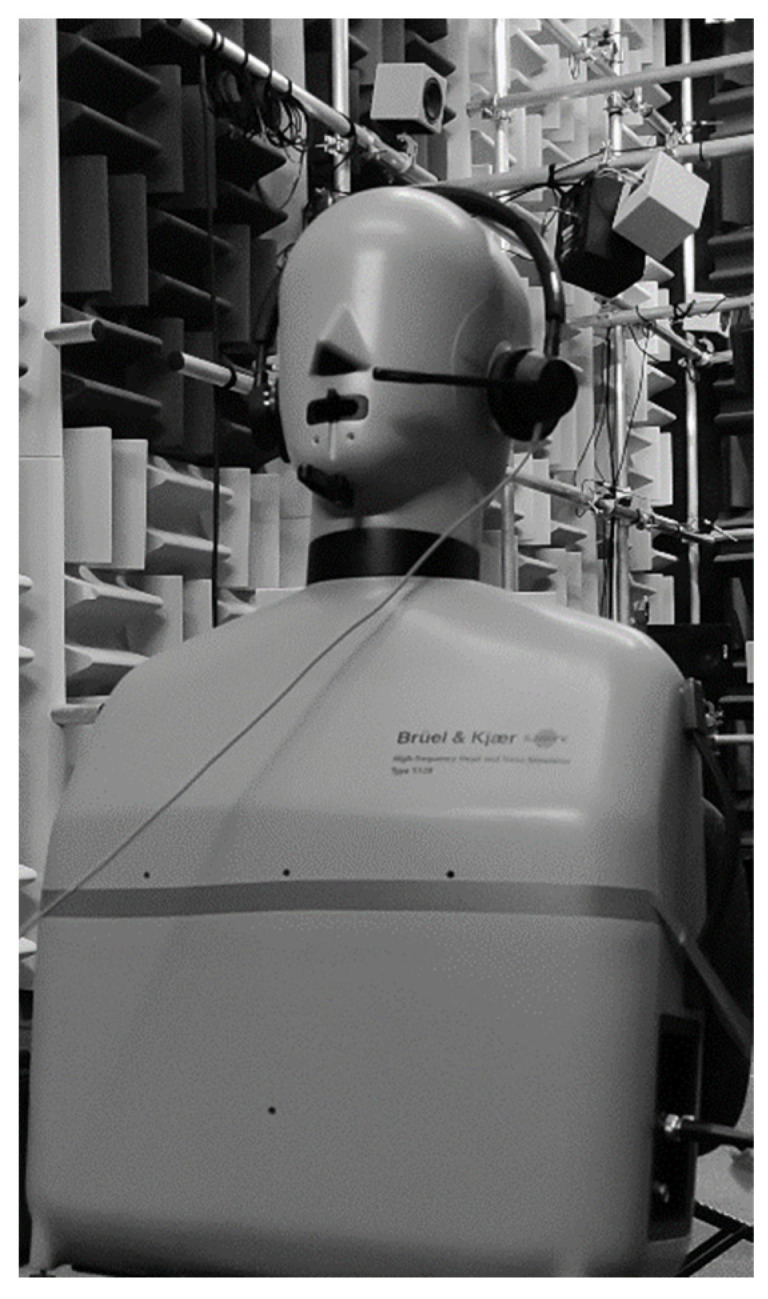
Headphones on high-frequency head and torso simulator.

A common aspect of many works aimed at testing sound parameters related to the use of headphones is the use of head and torso simulators for measurements [9,10,12,15,16,17]. In this study, a Brüel & Kjær 5128-C high-frequency head and torso simulator (HATS) (Hottinger Brüel & Kjær A/S, Virum, Denmark) was used to measure noise parameters. The HATS is designed to perform in situ electroacoustic tests on devices such as headphones, smartphones, headsets, conference phones, and hearing aids. This simulator makes it possible to perform tests in the full range of frequencies considered to belong to the audible range, which exceeds the capabilities of standard head-and-ear simulators, the required parameters of which are specified in a number of standards [18,19,20,21,22]. A picture of the HATS with headphones on during tests is shown in Figure 2. A Brüel & Kjær PULSE 3052-A-30 LAN-XI input module (Hottinger Brüel & Kjær A/S, Virum, Denmark) was used for testing. The analysis of measurement data was performed using the BK Connect 2019 software dedicated to the PULSE system, and Brüel & Kjær PULSE LabShop software. The measurements were carried out in an acoustic chamber at the Tech-Safe-Bio Laboratory (Central Institute for Labour Protection, National Research Institute, Warsaw, Poland).

### 2.3. Test Signal

The frequency responses of electronic systems, including electroacoustic systems, are normally tested using sinusoidal signals. If the electrical signal is measured at the sound card output, the test signal is a sinusoidal signal played back by the system sound player in the device. In this study, the test signal included 31 sinusoidal signals with frequencies covering the range considered to be audible, i.e., from 20 Hz to 20 kHz. Individual frequencies of the test signal generated for the tests were determined in accordance with the recommended EN 60318-1 standard [18]. A Brüel & Kjær 3160-A-042 output generator module (Hottinger Brüel & Kjær A/S, Virum, Denmark) was used for this purpose. The generated test signal was recorded in the form of a digital file intended for playback on the six devices included in the tests. Using the generated test signal, the frequency response at the electrical output of the device to which the headphones were connected was tested.

The generated sinusoidal test signal was also used in the acoustic tests through the measurement using microphones built into the high-frequency head and torso simulator. After determining the A-weighted equivalent sound pressure level associated with the use of headphones connected to the sound card integrated with laptop 1 (with CD player), another analogous series of measurements was carried out, except for the test signal used. In order to better represent the real use of headphones during the measurements, they were used to play back four types of sound: a children’s story on audiobook, a heavy metal song, a rock song, and a classical music piece.

### 2.4. Conversion of Results and Method of Analyzing Measurement Data

**Figure 3:**
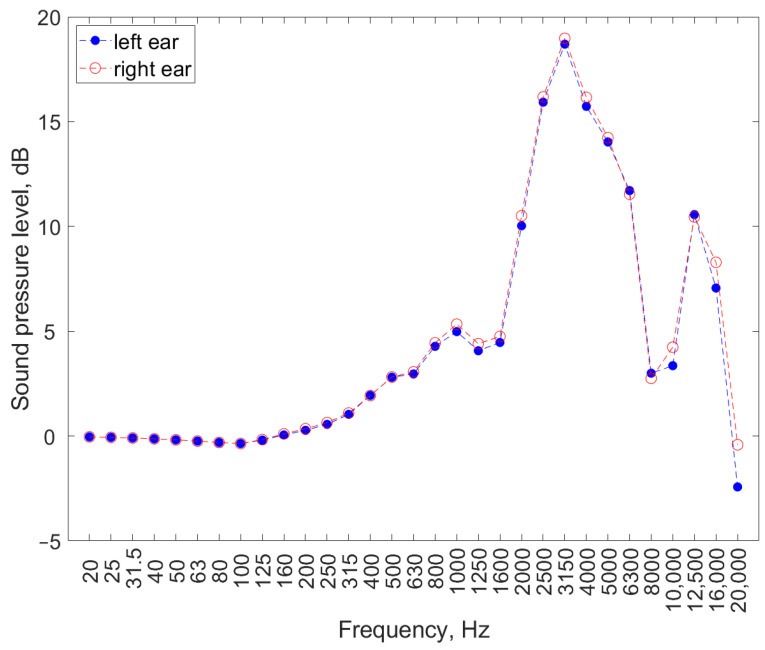
Listener’s free-field frequency response of head and torso simulator used in tests based on manufacturer’s data.

The results of the measurements using the head and torso simulator must be converted into the corresponding values in the acoustic field, which has been performed in similar studies [9,10,12,15], or in studies where the measurement was carried out directly in the human ear [8]. This conversion helps in using the results to assess noise parameters, according to the criteria for assessing noise exposure related to the work environment [5]. The conversion can be performed using standardized data [23] or data related to a specific head simulator, if available. For this purpose, the manufacturer’s data were used in this paper (Figure 3).

The criterion value regarding the A-weighted noise exposure level normalized to an 8 h working day is 85 dB, as mentioned in the introduction [5]. Maintaining the specified value, while increasing the A-weighted equivalent sound pressure level by 3 dB, requires a two-fold reduction in the exposure time, assuming that the criteria value related to the two further noise parameters are taken into account in the evaluation (i.e., the C-weighted peak sound pressure level (135 dB) and A-weighted maximum sound pressure level (115 dB) [5] are not exceeded). Assuming that the A-weighted equivalent sound pressure level to which a person is exposed was increased by 3 dB, it would amount to 88 dB; this means that in order to maintain the value of the A-weighted noise exposure level normalized to an 8 h working day at 85 dB, the exposure time should be reduced by half, i.e., to 240 min. Based on determining the A-weighted noise exposure level normalized to an 8 h working day, it is possible to determine the exposure time that cannot be exceeded, so as not to exceed the criterion value of 85 dB [24].

As mentioned in the introduction, the Commission Decision 2009/490/EC [4] recommended that European standards include the requirement to limit the sound level of personal music players, in order to prevent hearing damage. Since the value of the A-weighted equivalent sound pressure level amounted to 80 dB—which is considered not hazardous to hearing according to the SCENIHR report [3] and the Decision 2009/490/EC [4], and is in line with data from the standard with regard to determining occupational noise exposure and estimating hearing damage caused by noise [6]—the measurement results obtained for the audio pieces were evaluated using that criterion value of 80 dB.

## 3. Results

### 3.1. Measurements under Theoretical Conditions

**Figure 4:**
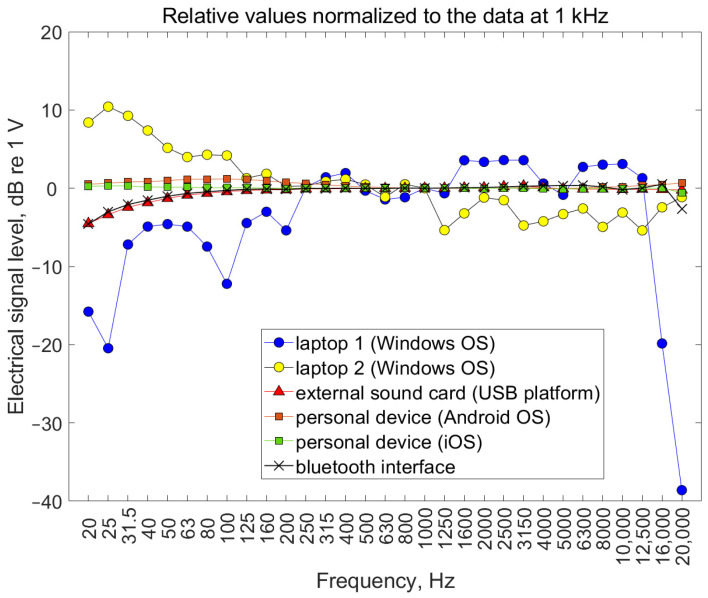
Level of electrical signals measured at electrical outputs of sound cards.

The results of electric signal measurements at the outputs of the sound cards of individual devices are presented in Figure 4. The main conclusion drawn from these diagrams is that for sound cards integrated with computer motherboards (of laptops), there is a significantly unequal shape of the frequency response of the signal fed to their output, (reaching 15 or 23 dB (1 V reference value)), compared to the personal devices. The improvement in the case of laptops may be due to the use of an external sound card, whose characteristics at low frequency ranges also decrease; however, the inequality does not exceed 5 dB (1 V reference value) and is monotonic along with the changing frequency of the signal. This is similar for wireless headphones that use a Bluetooth standard to transmit data. It is worth noting that in the case of high frequencies, all devices transmit sound signals without limitation, except for the sound card built into one of the laptops. The last 1/3 octave frequency band transmitting the sound had a center frequency of 12,500 Hz. Therefore, the sound signal transmission does not cover bands with frequencies of 16 and 20 kHz. This observation is important, since a limitation in the frequency band of the signal transmitted through the sound card will affect the sound playback, regardless of the quality of the headphones connected to the output of the card.

**Figure 5:**
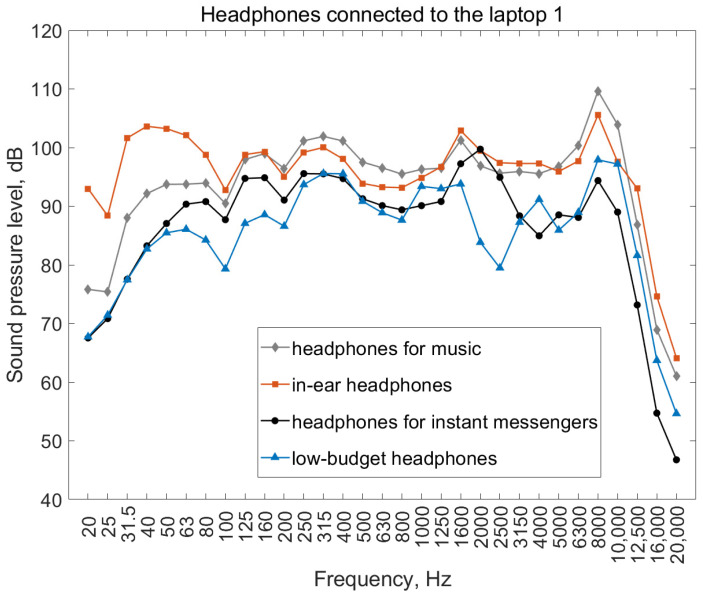
Sound pressure level measured at output of signal playback strip over headphones, taking into account transfer function of head and torso simulator (acoustic field).

The results of sound pressure level measurements, taking into account the transfer function (data of the head and torso simulator manufacturer; Figure 3), are presented in Figure 5.

The determined values of permissible headphone use time are presented in Table 1. The obtained values indicate that the time of playing back sound over headphones operating at theoretically the maximum capability, in the worst-case scenario, would have to be limited to less than one minute. Certainly, acoustic signals encountered in real life do not cover the full frequency band in the same way the the frequency response of the signal playback strip was examined, and high sound pressure levels do not occur continuously but only at certain time points. However, it is important to be aware of the hearing hazard potentially posed by the use of headphones.

### 3.2. Measurements in the Presence of Sounds Played Back in Real Life

Table 2 presents the results of measuring the A-weighted equivalent sound pressure level related to listening to music using headphones connected to a portable computer (laptop 1). The signal gain with the system adjustment slider was set to 100%. Table 2 also specifies the determined values of permissible listening time for specific types of sound attributable to an 8-h workday. The values were calculated in the same way as the data provided in Table 1, except that the criterion value was changed from 85 to 80 dB. The value of the A-weighted equivalent sound pressure level related to the use of headphones ranges from 75.2 dB, measured during playback of an audiobook with a children’s story, to 85.4 dB, measured during playback of music.

The determined values of permissible headphone use time show that among the pieces included in the tests, it is safest to listen to an audiobook. The permissible audiobook listening time at the 80 dB criterion exceeds 5 h, and when using headphones that produce the lowest A-weighted equivalent sound pressure level, the book content could be played 24/7. In the worst case scenario in terms of hearing exposure, the headphone use time should be limited to just over 2 h so that listening to rock music can be considered safe.

## 4. Discussion

The measurements were used to obtain information on the selected properties of signals converted from digital to analogue form by sound cards (electrical signal) of common-purpose devices, and the sound pressure level (acoustic signal) reaching the person using headphones. In the case of acoustic signals, measurements were made of the potentially largest possible sound pressure levels when using headphones. The electrical and acoustic properties of the signals will allow us to find solutions in the future in terms of the possibility of using them to play back sounds. The obtained numerical data and the conclusions drawn from them may be relevant when using applications that involve sound playback.

Regardless of how such applications are implemented and how they present sounds, it is essential to ensure safe conditions for their use. Naturally, measurements checking the parameters of sound potentially reaching listeners can be verified after the application is prepared, even before it is disseminated. Knowledge of the expected sound level associated with the use of headphones when working with a particular application will allow this to be checked in advance, so that using it does not create a risk of hearing damage. Applications of this kind, regardless of whether they are to be used at home (e.g., apps that support people with disabilities) or at work (e.g., as a part of occupational health and safety education), will not be used in laboratory conditions with supervised laboratory equipment, but will be used with common-purpose devices. Therefore, knowledge of the characteristics of signals played back using a laptop and generally available headphones can be useful in preparing software to achieve the intended practice and training purposes.

Much of the data on assessing the noise parameters associated with the use of headphones were collected prior to the publication of the previously mentioned Commission Decision [4]. The current results can therefore be compared with the historical data. According to data from the SCENIHR report [3], the A-weighted sound pressure level produced by personal players is within the range of 80 to 115 dB. The measurements carried out in the current work allowed us to check what values are related to the use of a laptop sound card and headphones a dozen or so years from the publication of Commission Decision 2009/490/EC [4]. The lowest A-weighted equivalent sound pressure levels are related to children’s stories in the form of an audiobook (from 75.2 dB), and the highest to music (85.4 dB). The difference between the four models of headphones for the individual types of sound is 3.5 to 5.3 dB. In general, the measured values of the A-weighted equivalent sound pressure levels are lower than those reported by SCENIHR [3] in 2008, with the highest values differing by almost 30 dB. The SCENIHR data concerned personal music players; however, both the SCENIHR report and the current study used headphones.

Another example is a paper reporting a study that measured sound pressure level generated by headphones available on the market for personal compact disc players [15]. As in this paper, that paper used a head and torso simulator, but it was a Knowles Electronics Manikin. Different types of headphones were considered: in-ear, supra-aural, and circumaural. The A-weighted equivalent sound pressure level, referring to the free-field, measured at the maximum volume setting, ranged from 91 to 121 dB. According to the authors, reasonable guidelines would include a recommendation to limit the use of headphones to 1 h or less per day in the case of supra-aurals, with the gain adjustment set to 60% of the maximum noise level. Therefore, the data confirmed the previous conclusion that currently available solutions have a lower A-weighted sound pressure level of real sounds being played back. Other, relatively newer data [12] show that the currently measured equivalent sound pressure levels assume values that do not exceed 100 dB. The lower A-weighted equivalent sound pressure level measured in this paper for real-life sounds (audiobook, musical pieces) translates into longer allowable use time for headphones containing sound playback strips. Therefore, this paper suggests that in the worst-case scenario, with the volume control set to 100%, that time is more than double (2 h and 18 min) compared to the 60% volume (1 h) suggested in the aforementioned paper [15].

In addition, also among the available historical data, an example showing that for the majority of headphones users, listening to music does not significantly increase risk of hearing loss, can be found [16]. In the referenced paper, measurements were made among 55 people who used personal stereos as part of their daily activity in conditions that could be considered loud, with an average daily noise exposure level set at 79.8 dB. However, as many as 25% of individual results had a value exceeding 85 dB, which resulted in the statement that a quarter of users of personal players are at risk of negative effects of noise.

The trend of limiting loud audio playback in headphones indirectly results from the combination of the highest A-weighted sound pressure levels set by people listening to rock music using in-ear headphones at 106.1 dB [17], with the theoretical in-ear headphone capacity considered in the present paper. The A-weighted equivalent sound pressure level of the test signal, at the highest possible volume at each measurement frequency that was constant over time, exceeded the referenced value of 106.1 dB only by 4.6 dB (data from Table 1). It should be emphasized that the value taken from the literature [17] did not refer to the theoretical frequency response of headphones, but resulted from measuring the noise parameter during music playback. For the sake of comparison, reference should be made to the value of 85.2 dB in this paper (data from Table 2) related to listening to a rock song with the use of in-ear headphones controlled by a computer sound card with the volume set to 100%.

The results of measurements made in the presence of real-life sounds indicated that in the worst case in terms of hearing safety, i.e., rock music, it would be possible to listen for 2 h and 18 min. It should be noted that the measurements related to the use of headphones playing various types of sound were carried out with the gain in the music playback strip set to 100%. The permissible music-listening time was determined assuming continuous exposure to sound (in this case, music). It can therefore be concluded that the use of headphones connected to a common-purpose device, such as a portable computer, without using the maximum settings for gain adjustment or playing the music continuously, can be considered safe for a few hours per day. The indicated time limit for the safe use of headphones, in the case that is least favorable for hearing, results from the A-weighted equivalent sound pressure level of 85.4 dB. It can be assumed that listening to music in practice would involve significantly lower sound parameter values. This can be presumed, taking into account that a sound pressure level of 71.6 dB is considered medium or comfortable and 97.8 dB very loud [25]; this is a difference of 26.2 dB. The output level measured in the current work was significantly lower than the level characterized as very loud in the quoted paper [25]. Reducing the A-weighted equivalent sound pressure level measured in this paper by 5.4 dB, i.e., to 80 dB, would make it possible to use headphones in this way for 8 h per day.

The difference between the A-weighted equivalent sound pressure levels of four tested headphone models, depending on the type of sound used as the test signal (Table 2), ranged from 4.9 dB (classical music) to 7.4 dB (heavy metal). In the literature, the data usually indicate that in-ear headphones result in a higher sound level than other types of headphones, e.g., 7–9 dB [15]. In this paper, lower sound levels are associated with the use of low-budget, circumaural, closed-back headphones and supra-aural headphones for instant messenger. The highest A-weighted equivalent sound pressure level was measured in the case of in-ear headphones, but slightly lower or comparable values were observed with headphones for music (circumaural, open back). The latter are dedicated to users who appreciate high-quality sound and have relatively high efficiency. 

The four models considered in the study were closed-back, open-back (2 models), and in-ear headphones. In one study it was found that using in-ear and closed-back headphones may be related to hearing loss at higher frequencies [13]. It was also reported that open-back and semi-open headphones are safer because they do not cause hearing damage. However, when considering the results obtained in the current paper, it is not always possible to associate the type of headphone structure (closed-back, open-back, in-ear, etc.) with any effect on hearing. The measurement results (Table 2) indicate that despite the fact that two models (low-budget and headphones for instant messengers) differ in their structure, i.e., one is closed-back and the other is open-back, the values of sound parameters measured when using them were relatively similar and significantly different from those for the other two models, open-back and in-ear. In the case of the closed-back and open-back headphones (low-budget and for instant messengers), the A-weighted equivalent sound pressure levels ranged from 75.2 to 81.7 dB, regardless of the type of sound being played back. However, in the case of the open-back (for music) and in-ear headphones, the values ranged from 81.4 to 85.4 dB. It can, therefore, be seen that the noise parameter values are determined not only by the design but also by other features of the headphones.

The limitation of this work is the number of devices included in the electrical signal measurements, which was six, and the number of headphones included in the acoustic signal measurements, which was four. However, the aim was not to characterize devices available on the market, but to verify the correctness of various types of equipment. It was important to prioritize the type of equipment according to the faithful transmission of sound as a function of the signal frequency. In this case, a personal device such as a smartphone, an external sound card with a USB interface, and wireless headphones with Bluetooth interface tended to be the best. Sound cards built into laptops have the worst characteristics in terms of the frequency response of transmitted electrical signals. Worse still, in the case of a sound card built into the laptop, it is also possible to narrow down the upper range of the useful frequencies of the signal being transmitted. This effect most likely had an adverse impact on the measurement of the acoustic signal characteristics of the four types of headphones tested. In such a situation, knowledge of the band of the signal transmitted through the headphones does not give a complete picture as such, as it is important to know the characteristics of the entire sound transmission strip. Hence, in this work, we intentionally did not take into account the characteristics of headphones as standalone objects, but examined the signal properties at the end of the entire sound playback strip. 

As was already mentioned, a limitation of the transmitted frequency band, resulting in relative reduction in the sound level at 1/3 octave bands of 16 and 20 kHz, could potentially also impact the final A-weighted equivalent sound pressure level. These values do not correspond to the situation in which the transmitted frequency band of the transferred frequency does not have hardware limitations. In order to analyze this problem, additional calculations were carried out, in which it was assumed that the characteristics of the sound playback strip (Figure 5) within the high-frequency range did not decrease. Ignoring the reason for the reduced frequency response, i.e., whether it resulted from the imperfection of the laptop sound card or the headphones themselves, it was assumed that the sound pressure level at frequencies of 12.5, 16, and 20 kHz was the same as that at 10 kHz. 

In the case of circumaural headphones of relatively good quality intended for music playback, at theoretical full control (Table 1), this means changing the A-weighted equivalent sound pressure level from 112.2 to 112.7 dB, which reduces the theoretical permissible use time from 54 to 48 s. However, the same calculations carried out for real-life sounds showed that the A-weighted equivalent sound pressure level did not change when the sound source was an audiobook (81.4 dB), a heavy metal song (83.2 dB), a rock song (85.4 dB), or a piece of classical music (82.9 dB). In these four situations, the results differed only in the second decimal place. The relatively small changes, or lack of change, in the A-weighted equivalent sound pressure level occur because the frequency-weighting A takes into account high- (and low-) frequency sounds with less weight than sounds from the center of the audible frequency band. Therefore, limiting the high-frequency signal by the sound card will result in an inaccurate representation of the sound; however, this will not have a significant impact on the results of assessing noise exposure.

## 5. Conclusions

The measurements showed that the most uniform shape, as a function of frequency, of signal characteristics at the electrical outputs of sound playback devices was in personal devices, regardless of the operating system (Android, iOS). For sound cards integrated with laptop motherboards, there is a significantly unequal shape of the frequency response of the signal fed to the output, with the inequality reaching 15 or 23 dB, compared to personal devices. In the case of one of the laptops, the upper frequency range was limited to 1/3 octave band of 12,500 Hz. The improvement in the case of laptops may be due to the use of an external sound card, whose characteristics in the low-frequency range also decrease; however, the inequality does not exceed 5 dB. A similar improvement may take place when using wireless headphones that transmit data using Bluetooth.

Tests carried out on audio strips consisting of four headphone models and a sound card integrated into a laptop, representing various practical solutions, showed that if these strips were used at the theoretical continuous maximum, in order to protect hearing, their use should be limited to just a few minutes. If the test signal includes real-time sounds, e.g., audiobook content or music, in the case that is the least favorable in terms of hearing safety (rock music), the listening time can be 2 h and 18 min.

The cases considered in the analysis show that for training systems or systems that convey important audio information, the choice of the hardware platform may be relevant when it is necessary to accurately reflect sounds. The A-weighted equivalent sound pressure level associated with the use of headphones has generally decreased compared to the data reported several years ago; nevertheless, studies on developing systems to protect the hearing of the people using them should include an assessment of the parameters of the sounds that are played back.

## Figures and Tables

**Table 1:** Permissible time of exposure to sound played back over headphones connected to laptop 1 in theoretical case of extremely high volume (signal with greatest possible level of control at each measurement frequency, at a constant level over time), determined with an assumed criterion value of A-weighted noise exposure level, normalized to an 8 h working day of 85 dB.

Parameter	Low-Budget Headphones, Circumaural, Closed-Back, with Microphone on Cable, Approximate Price EUR 15	Headphones for Instant Messengers, Supra-Aural, Open-Back, with Microphone on Wire, Approximate Price EUR 65	Headphones for Music, Circumaural, Open-Back, No Microphone, Approximate Price EUR 150	In-Ear Headphones, with Microphone on Cable, Approximate Price EUR 9
L_Aeq_, dB ^1^: theoretical values resulting from measurement of frequency response	103.7	105.5	112.2	110.7
Theoretical permissible headphone use time	388 s (6 min 28 s)	256 s (4 min 16 s)	54 s (0 min 54 s)	77 s (1 min 17 s)

^1^ L_Aeq_, A-weighted equivalent sound pressure level.

**Table 2:** Permissible exposure time to sound played back over headphones connected to laptop 1 when listening to real-life recordings (signal varying with time, limited spectrum according to content of recording), determined with an assumed criterion value of 80 dB, which is considered not to pose a risk of hearing damage.

Headphones	Test Signal/Piece	L_Aeq_ (dB) ^1^	Permissible Headphone Use Time		
			h	m	s
Low-budget, circumaural, closed-back, with microphone on cable, approximate price EUR 15	audiobook	75.2	24	9	34
	heavy metal	77.0	15	57	43
	rock	79.9	8	11	10
	classical	78.0	12	40	44
For instant messengers, supra-aural, open-back, with microphone on wire, approximate price EUR 65	audiobook	76.4	18	19	36
	metal	80.2	7	38	23
	rock	81.7	5	24	31
	classic	78.7	10	47	30
For music, circumaural, open-back, no microphone, approximate price, EUR 150	audiobook	81.4	5	47	43
	metal	83.2	3	49	44
	rock	85.4	2	18	26
	classical	82.9	4	6	10
In-ear, with microphone on cable, approximate price EUR 9	audiobook	81.7	5	24	31
	metal	84.4	2	54	16
	rock	85.2	2	24	57
	classical	82.9	4	6	10

^1^ L_Aeq_, A-weighted equivalent sound pressure level.

## Data Availability

All data are stored digitally by the researcher.
